# Diesel exhaust particles modulate the tight junction protein occludin in lung cells *in vitro*

**DOI:** 10.1186/1743-8977-6-26

**Published:** 2009-10-08

**Authors:** Andrea D Lehmann, Fabian Blank, Oliver Baum, Peter Gehr, Barbara M Rothen-Rutishauser

**Affiliations:** 1 Institute of Anatomy, University of Bern, Bern, Switzerland; 2 Department of Clinical Research, University of Bern, Bern, Switzerland

## Abstract

**Background:**

Using an *in vitro *triple cell co-culture model consisting of human epithelial cells (16HBE14o-), monocyte-derived macrophages and dendritic cells, it was recently demonstrated that macrophages and dendritic cells create a transepithelial network between the epithelial cells to capture antigens without disrupting the epithelial tightness. The expression of the different tight junction proteins in macrophages and dendritic cells, and the formation of tight junction-like structures with epithelial cells has been demonstrated. Immunofluorescent methods combined with laser scanning microscopy and quantitative real-time polymerase chain reaction were used to investigate if exposure to diesel exhaust particles (DEP) (0.5, 5, 50, 125 μg/ml), for 24 h, can modulate the expression of the tight junction mRNA/protein of occludin, in all three cell types.

**Results:**

Only the highest dose of DEP (125 μg/ml) seemed to reduce the occludin mRNA in the cells of the defence system however not in epithelial cells, although the occludin arrangement in the latter cell type was disrupted. The transepithelial electrical resistance was reduced in epithelial cell mono-cultures but not in the triple cell co-cultures, following exposure to high DEP concentration. Cytotoxicity was not found, in either epithelial mono-cultures nor in triple cell co-cultures, after exposure to the different DEP concentrations.

**Conclusion:**

We concluded that high concentrations of DEP (125 μg/ml) can modulate the tight junction occludin mRNA in the cells of the defence system and that those cells play an important role maintaining the epithelial integrity following exposure to particulate antigens in lung cells.

## Background

With every breath millions of ambient particles enter the respiratory tract, where they may cause adverse health effects associated with increased pulmonary and cardiovascular mortality [1-4]. Important components of ambient particulate matter (PM) are diesel exhaust particles (DEP), which are comprised of a carbon core that absorbs a mixture of metals and organic chemicals [5]. Nearly all these particles have sizes less than 1 μm, and the majority of those particles are known as ultrafine particles with a diameter less than 0.1 μm [6]. Due to the small size of DEP, they can even reach the bronchiolar and alveolar levels of the lung and up to 33% of the inhaled particles are deposited in the respiratory tract [7]. In several *in vitro *studies, it was shown that DEP can penetrate into the epithelial cells (16HBE14o-) [8,9] and that inhalation of high concentrations of DEP affect respiratory function in humans (see review [10]). Therefore the airway epithelial cells are directly affected by air pollution and may play a key role in the pathophysiology of airway diseases.

Among several structural and functional barriers which protect the respiratory system against PM (for a review see [11,12]), epithelial cell layers endowed with tight junctions (TJ) [13,14] are one of the most important. TJ separate the epithelium in an apical and a basal site and control the paracellular transport, by preventing macromolecules from easily passing through the epithelial layer for example. In addition, a population of macrophages in the airways and in the alveoli [15,16], and a network of dendritic cells (DC) underneath and inside the epithelium [17-19] are other important parts which contribute to the functionality of the barrier.

Of particular importance and interest is how inhaled particulate antigens come into contact with DC, which realize, as sentinels and most competent antigen presenting cells, a surveillance network in the pulmonary tissues [19-21]. The mechanism of how DC can penetrate TJ of the epithelium is not completely understood [22]. Studies with mice [23] and with an *in vitro *model of the gut epithelium [24,25] have shown that DC express TJ proteins and it was assumed that DC build TJ-like complexes with epithelial cells in order to preserve the epithelial integrity. Using an *in vitro *triple cell co-culture model of the human airway wall [26,27] it was recently shown that human blood monocyte-derived dendritic cells (MDDC), as well as human blood monocyte-derived macrophages (MDM) express TJ proteins and mRNA (occludin, Claudin-1, zonula occludens 3 (ZO-3), junctional adhesion molecule 1 (JAM-1) and the Adherens Junctions (AJ) mRNA and protein E-cadherin) [28]. Moreover it has been shown that processes of DC residing under the epithelium can penetrate beyond well developed epithelial TJ to sample particles and to transport them to the basal side of the epithelium [24,25,27,29,30].

As previously published, large particles can be sampled by the cells of the defence system and therefore overcome the epithelial airway barrier. This has also been shown for nanosized particles (NP) in the triple cell co-culture system, although the translocation characteristics of fine particles compared to NP was different [31]. Furthermore it has been demonstrated that inhaled NP quickly left the lungs and were detected in the extra-pulmonary tissue [32,33].

This data emphasizes the importance to study the effect of airborne PM on the epithelia and the epithelial integrity. If there are any alterations in the integrity of the epithelial barrier more attention has to be focused on the fate of inhaled nanosized, as well as larger particles, because the epithelia is not able to fulfil its barrier function. Following cigarette smoke exposure to Calu-3 cells, amongst others, it was found that there was a displacement of occludin and Zonula occludens 1 (ZO-1) and thus increasing ionic and macromolecular permeability. It was hypothesized that the loss of TJ integrity and barrier function was due to a decreased ZO-1: occludin association ratio [34].

The aim of the present study was to determine the extent to which DEP affect the integrity of the airway wall, by focussing on the expression of the TJ protein occludin in epithelial cells (EC), MDM and MDDC (which all contribute to the integrity of the airway wall as previously mentioned) after exposure to different amounts of DEP. The plasticity alteration of TJ is known [35], therefore the mRNA and protein levels of the occludin were investigated for changes, upon particle exposure. For this purpose mono-cultures of MDM and MDDC as well as of the bronchiolar cell line 16HBE14o- were used and exposed to different concentrations of DEP particles or to the inflammatory stimulus Lipopolysaccharide (LPS) for 24 h. Transepithelial electrical resistance (TEER) was measured in the EC mono-cultures and in the triple cell co-cultures. Cytotoxicity was assessed by Lactate dehydrogenase (LDH) measurement and the pro-inflammatory cytokine tumor necrosis factor alpha (TNFα) was determined by ELISA. The spatial localisation of occludin in the different cell types was investigated by laser scanning microscopy (LSM) and the TJ arrangement by transmission electron microscopy (TEM).

## Results

### DEP is taken up by all cell types

**Figure 1:**
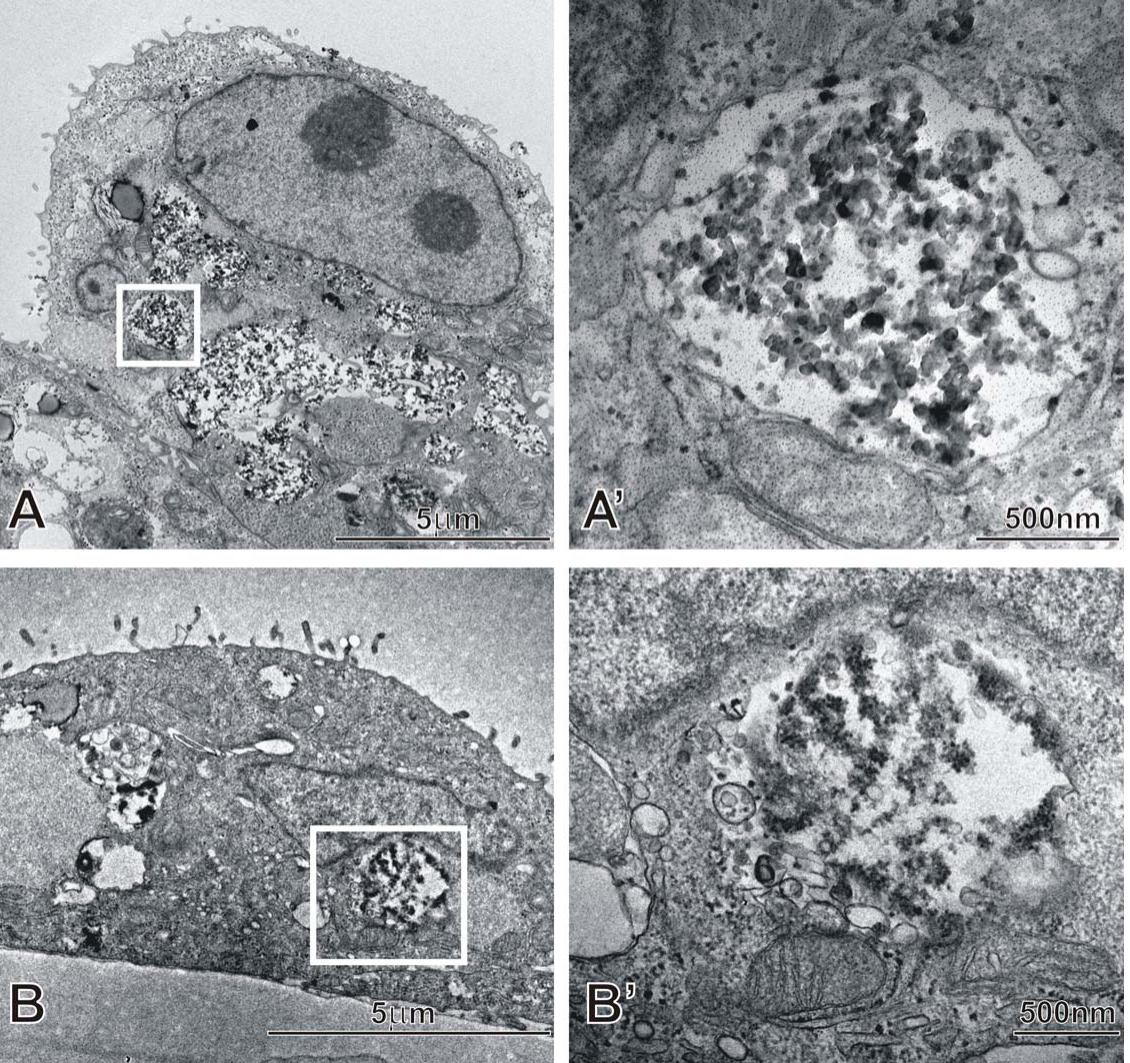
**Internalized DEP in an epithelial cell**. TEM images of 16HBE14o- cells in the triple cell co-culture model showing internalized DEP. Cells were exposed to 125 μg/ml DEP (A) and 0.5 μg/ml DEP (B) for 24 h and then processed for TEM. A detailed view from A and B (white square) is shown in A'and B'.

In a first step, the DEP exposed cells were studied by TEM. As shown in Fig. 1, DEP (125 and 0.5 μg/ml) could clearly be seen in membrane bound vesicles inside EC in the triple cell co-culture system independent of the dose used, indicating that DEP was taken up by the cells. Cells exposed to 125 μg/ml showed higher amounts of internalized DEP, however this was only a qualitative observation and was not quantified. DEP was found in all investigated cell types in mono-cultures (shown in this study) as well as in the three cell types of the triple cell co-cultures, as already shown in a recently published paper from our group [36].

### TJ between ECs and TJ-like structures between EC and MDM

**Figure 2:**
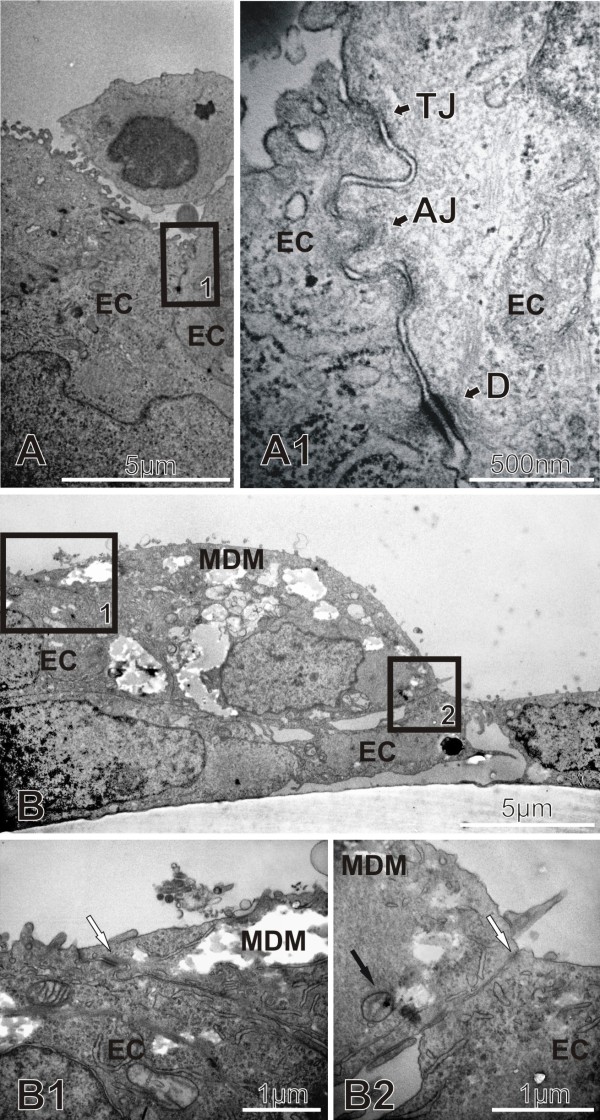
**TJ between EC and between EC and MDM**. TEM images showing untreated cells in the triple cell co-culture model in A. The tight junctions (TJ) the adherens junctions (AJ), as well as the desomosomes (D) between two neighbouring EC are shown in the detailed view in A1. TJ-like structures (white arrows, B1 and B2) between a MDM and two neighbouring EC in the triple cell co-culture model is shown in overview in B. B1 and B2 are detailed views. The morphology and as well as the internalized gold NP (black arrow in B2) prove that the described cell is a MDM.

The cell-cell junctions in 16HBE14o- cells are shown in Fig. 2A. The junction complex consisting of desmosomes, AJ and TJ are well defined as determined with TEM. In addition, it was also shown with TEM that EC and MDM build TJ-like complexes. Since MDM are very difficult to find with TEM in the triple cell co-culture system, the MDM were exposed to gold NP before they were added to the epithelial cells. In Fig. 2B the MDM has clearly been identified by intracellular, membrane-bound gold particles (Fig. 2B2, black arrow). Cell junctions similar to the ones seen between EC were identified (Fig. 2B1, white arrow).

### DEP exposure affects the amount and the distribution of occludin protein in EC, MDM, and MDDC

**Figure 3:**
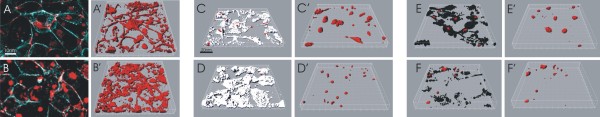
**Laser scanning micrographs of occludin in EC, MDM and MDDC**. The occludin arrangement in 16HBE14o-, MDM and MDDC mono-cultures visualized with LSM. Control 16HBE14o- cells (A) and DEP exposed 16HBE14o- cells (B) were labelled for F-actin (turquoise) and occludin (red). For F-actin no qualitative difference was found between control and treated cells. However, the iso-surface presentation of occludin in control cells (A') showed a more structured TJ occludin belt compared with DEP treated cells (B") which shows a predominant localization of occludin in the cytoplasm. Occludin was visualized in mono-cultures of MDM (D, control; C, DEP treatment) and MDDC (E, control; F, DEP treatment). Cells were stained for F-actin (in MDM white, in MDDC black and occludin (shown in red). MDM and MDDC expressed the TJ protein occludin. After exposure to DEP for 24 h occludin signals were reduced (for MDM in D' and MDDC in F'). These are representative images and for all pictures the same settings were used.

The arrangement of the TJ was analysed by focusing on the transmembrane protein occludin. Visualization was done using LSM and digital image restoration, as described in an earlier publication [30]. DEP treated (125 μg/ml) and untreated EC were stained for F-actin cytoskeleton and occludin (Fig 3A, B). The iso-rendering of occludin revealed an intact and clear belt-like structure of the TJ protein, occludin, in the untreated EC mono-cultures (3A'). Although occludin is detectable in the DEP treated mono-cultures, the belt like arrangement of the TJ is not as structured (Fig. 3B') as in the untreated mono-cultures (Fig. 3A'). The amount and the arrangement of F-actin were not affected by the DEP treatment (Fig. 3A, B). Mono-cultures of MDM and MDDC were also stained for occludin (Fig. 3C-F). The pictures clearly showed that after DEP treatment the amount of occludin was reduced in MDM (D') as well as in MDDC (F').

### DEP does not significantly affect the occludin mRNA expression in EC, MDM, and MDDC

**Figure 4:**
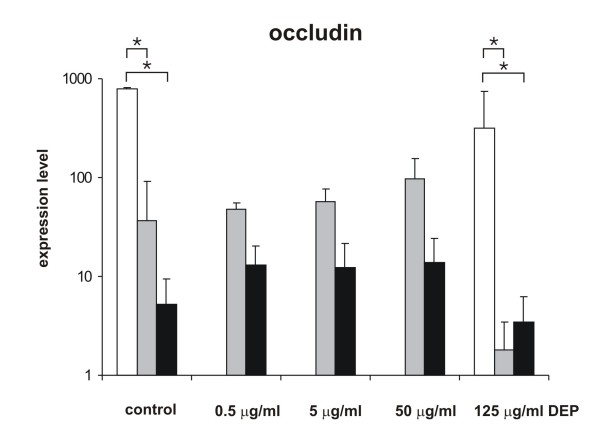
**mRNA expression levels of occludin in EC, MDM and MDDC**. Quantification of occludin mRNA expression in MDM, MDDC, and EC mono-cultures by qRT-PCR. Threshold of occludin was set by the threshold of the housekeeping gene β-actin in each sample (ΔCT). Expression levels of occludin are illustrated by the β-actin normalized concentrations (2^-ΔCT ^× 15000). cDNA derived from MDM (grey columns), MDDC (black columns) and 16HBE14o- (white columns) control cells and cells treated with DEP (0.5 μg/ml, 5 μg/ml, 50 μg/ml, and 125 μg/ml). Values are presented as means ± SD; * p ≤ 0.05. All experiments were independently performed between 4-7 times.

To examine if the mRNA level of occludin was quantitatively different after DEP treatment, the amount of mRNA in mono-cultures of EC, MDM and MDDC was determined using qRT-PCR. MDM and MDDC were exposed to different doses of DEP, ranging from low to high concentrations (0.5 μg/ml, 5 μg/ml, 50 μg/ml, and 125 μg/ml). EC were only exposed to the highest amount of DEP because preliminary experiments showed no changes upon exposure to lower DEP concentrations. Although the results were not significant, there was a tendency that the highest dose of DEP reduced the occludin mRNA level in MDM and MDDC (Fig. 4). The expression level of occludin in EC was significantly more than 100 times higher compared to MDM and MDDC, as also previously shown in [28].

### DEP exposure affects TEER in 16HBE14o- mono-cultures but not in triple cell-co-cultures

**Figure 5:**
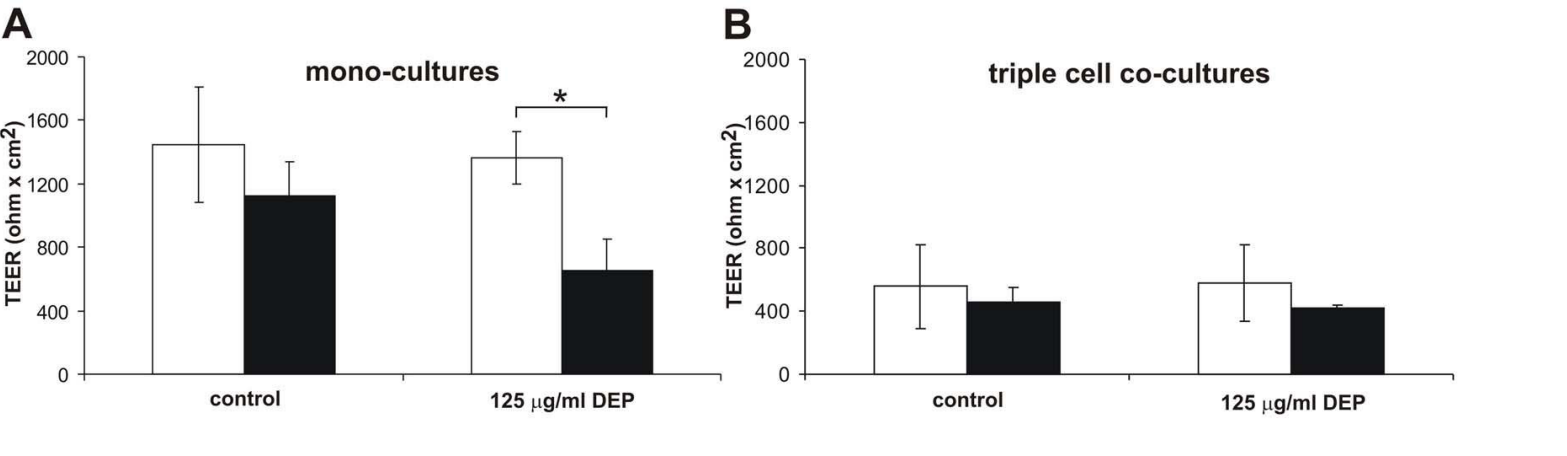
**Epithelial integrity of EC mono- and triple cell co-cultures**. TEER in16HBE14o- mono-cultures (A) and triple cell co-cultures (B) before (white bars) and after (black bars) exposure to high dose of DEP (125 μg/ml) or normal medium (control) for 24 h. TEER in mono-cultures was significantly lower after DEP treatment than before addition of DEP (A, *). Data are presented as means ± standard deviation (SD); * p ≤ 0.05. All experiments were independently done in triplicate.

To examine whether there was a change in the epithelial integrity in EC mono- and triple cell co-cultures, the TEER was measured before and after 24 h exposure to DEP (125 μg/ml). In mono-cultures, the TEER after DEP treatment was significantly reduced (p = 0.029) compared to control cultures, as shown in Fig. 5A. In triple cell co-cultures there was no difference in the TEER levels found between control and DEP treated cultures (Fig. 5B). The epithelial integrity of untreated triple cell co-cultures compared to the mono-cultures was significantly lower (p = 0.024) (Fig. 5).

### DEP exposure (of up to 125 μg/ml) does neither affect cytotoxicity nor TNFα levels in EC mono-cultures, and in triple cell co-cultures

**Figure 6:**
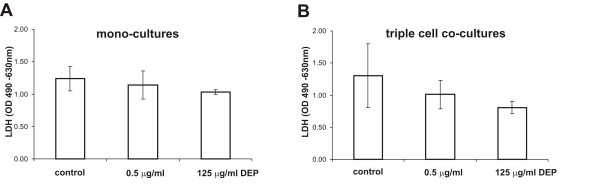
**Cytotoxicity of EC mono- and triple cell co-cultures**. LDH (a marker for membrane permeability) levels were determined. The values did not change significantly after DEP exposure in epithelial mono-cultures (A) and triple cell co-cultures (B). Data are presented as mean ± SD of 3 experiments.

To ensure that the reduced TEER in mono-cultures was not due to increased cell death the cytotoxicity of the highest DEP concentration was investigated by measurements of LDH release in the triple cell co-cultures and in EC mono-cultures. The difference of the LDH release into the cell culture medium was not significant between the controls and the DEP exposed cells (Fig. 6).

**Figure 7:**
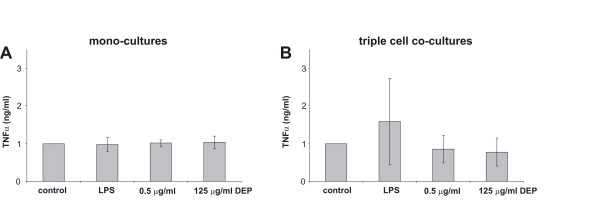
**TNFα release in EC mono- and triple cell co-cultures**. TNFα release in mono-cultures (A) and triple cell co-cultures (B) upon exposure to DEP for 24 h. TNFα concentrations were expressed as mean ± SD of 4-5 experiments. DEP treated EC mono-cultures and the triple cell co-cultures showed no difference in TNFα concentration in comparison to the respective untreated cells. No differences between mono-cultures and triple cell co-cultures concerning TNFα levels were found. LPS stimulated cultures were used for the positive control.

The pro-inflammatory response related to DEP exposure was measured by the TNFα levels in the supernatants from EC mono-cultures and from the triple cell co-cultures by ELISA (Fig. 7). There was no significant difference in the TNFα amount between control cultures and cells exposed to different DEP concentrations. LPS was used as positive control and resulted in an enhanced release of TNFα into the supernatants in the triple cell co-culture.

### Dynamics of TJ in the EC mono-cultures and triple co-cultures under EDTA treatment

**Figure 8:**
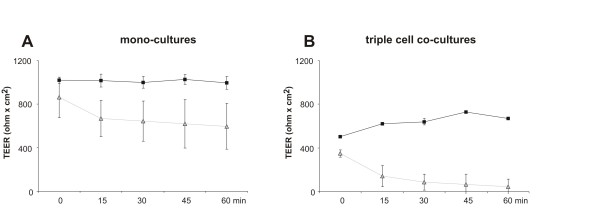
**Opening of the TJ in EC mono- and triple cell co-cultures**. TEER values after EDTA treatment (grey lines) over one hour in mono-cultures (A) and in triple cell co-cultures (B). Untreated controls are presented in black.

To test whether the absence of change in TEER, in the triple cell co-culture model, may be due to different exposure kinetics (i.e. that the cells in the 3-dimensional configuration may be less sensitive), a Ca^2+ ^chelator was used which is known to open the TJ. Adding Ethylenediaminetetraacetic acid (EDTA) as a Ca^2+ ^chelator, the TEER values of mono-cultures as well as those of triple cell co-cultures were reduced in a time-dependent manner (Fig. 8A and 8B). In the mono-cultures, as well as in the triple cell co-cultures, the TEER value decreased about 300 ohm × cm^2 ^per hour, showing the same TJ kinetics. The untreated control cultures showed no change in TEER.

## Discussion

It is known that DEP can negatively influence human health. However, the question is still unclear as to how inhaled DEP and other particulate matter can penetrate into the blood flow. At least it seems that the barrier which separates the air from the blood must be crossed. Therefore, the influence of DEP on the TJ protein occludin was examined. It was already shown that the TJ play a key role in the regulation of paracellular transport [34]. Without intact TJ arrangements the epithelial layer would not remain tight, and there would be leakage, allowing deposited particles an enhanced translocation across the barrier.

The effect of DEP exposure on the expression of the TJ protein and mRNA occludin was studied in mono-cultures of MDM, MDDC and the epithelial cell line. Moreover the epithelial mono-cultures of EC were compared to the triple cell co-culture model of the epithelial airway barrier, which has been previously described and characterized [26,27,37]. The triple cell co-culture model mimics the natural architecture of the *in vivo *epithelial airway and is accepted and used per se in several studies [26,30,37,38]. The cells of the defence system used in the triple cell co-culture model have similar functional and structural characteristics as the ones in human lung [39]. The bronchial epithelial cell line 16HBE14o- used in the present study, forms polarized monolayers with extensive TJ belts which were also verified with TEM technique. Therefore, this cell line is a useful tool to study TJ-properties, such as biogenesis, regulation and breakdown, which may in turn prove to be useful paradigms of the permeability functions of human bronchiolar epithelium, especially in studies of TJ assembly and disassembly [40,41]. Additionally the presence of intact TJ in this cell line was shown to release pro-inflammatory cytokines in response to DEP [8]. In a previous study, it was already shown that EC, and also MDM and MDDC, express the TJ proteins claudin-1, ZO-3, JAM-1 as well as the adherens junction protein E-Cadherin. [28] For an initial evaluation of the ability of DEP to influence the TJ, the current study primarily focused on the TJ mRNA and protein occludin and its cellular localization because it was shown that the extracellular domains of this protein are important for correct localization of occludin in the TJ-complex and for the regulation of the paracellular permeability barrier [42]. The influence of DEP on TJ proteins other than occludin will be the aim of further studies. As a surrogate for DEP, the standardized and characterized Diesel Particulate matter (SRM Nr. 2975; National Department of Standards and Technology, Gaithersburg, USA) were used. Similar concentrations as used in other studies [5,8,9,43,44] were applied but with the understanding that the highest used dose (125 μg/ml), exceeds doses which occur in realistic exposure scenarios. However, concentration series are important to determine if a threshold dose exists or not.

DEP were visualized inside EC of the triple cell co-culture model as well as in mono-cultures of EC, MDM and MDDC. This was in agreement with other studies which showed an intracellular localization of DEP in the EC line 16HBE14o- as well as in MDM and MDDC [8,36,45]. DEP aggregates were mostly found in membrane bound vesicles, but so far single DEP particles or smaller aggregates in the nucleus have not been detected with conventional TEM, as described in the study of Boland et al. [8]. It is very difficult to detect single DEPs or small aggregates with conventional TEM. Sophisticated TEM methods, such as electron energy loss spectroscopy, would be needed to detect specific metals associated with the DEPs.

The epithelial integrity in the triple cell co-cultures did not change after exposure to high concentrations of DEP (125 μg/mL). However, when EC mono-cultures were exposed to the same DEP dose the TEER was significantly reduced. In the triple cell co-cultures the total number of cells is slightly higher due to the additional MDM and MDDC. The number of MDM and MDDC is similar to the *in vivo *situation of the human lung [30]. It was hypothesised that this would lead to a lower DEP burden per cell. MDM which are positioned at the apical side of the epithelium [15,16,46] might phagocyte the main amount of deposited DEP, thus the DEP load to the EC layer in the triple cell co-culture model may be lower than that of the EC mono-cultures. This could also explain why, after exposure with the highest concentration of DEP, no effect on EC integrity in the triple cell co-culture model could be measured. This finding shows the importance of those professional phagocytes [46] and the significance of using more realistic co-cultures, such as the triple-cell co-culture model used in this study. However, the control EC as well as DEP exposed mono-cultures showed higher TEER compared to the triple cell co-cultures. The interactions of the MDM and MDDC within the epithelium may explain the lower TEER values. On the one hand the EC cells are probably less tightly connected in the EC layer due to the interactions with the cells of the defence system but on the other hand, the TJ-like structures between EC and MDM, MDDC may be able to prevent a leakage barrier. Such TJ like structures could be visualized with TEM. Due to the morphology, the position on the EC layer and the intracellular gold NP it was possible to define the cell in the triple cell co-culture model as MDM, which make TJ-like junctions with the two neighbouring ECs. Recently it was shown that MDM and MDDC are able to interact in the triple cell co-culture as a transepithelial network, by building cytoplasmic processes (with their pseudopodia) between EC, which could lead to an opened TJ-belt of the epithelial cells. However, since this study has shown that MDM and MDDC would build TJ-like structures with the EC, the integrity would remain, although maybe the tightness is decreased to a certain level. This seems to be an important mechanism for cells penetrating the epithelial cells, to keep the tightness of the cell layer although maybe to a reduced level. The addition of polystyrene particles did not change the epithelial integrity, which was also the same in the present study when DEP were added [30]. The present findings are in contrast to the results of the study published by Bayram and co-workers [5], who demonstrated that after exposing cultured human bronchial epithelial cells to concentrations of DEP between 10-100 μg/ml the TEER increases significantly but without effect on the passage of ^14^C-bovine serum albumin across the cell culture [5]. It is important to note that it is difficult to compare different studies due to the use of different types of DEP and cell cultures.

The decrease in barrier function in the EC mono-cultures can not be explained by cell death. No change in cytotoxicity was found, by determination of LDH levels in the supernatants, after exposure to 125 μg/ml for 24 h. This is in agreement with a study done by Boland and co-workers [9]. That study found a cytotoxic effect in 16BE14o cells exposed either with standard particles (SRM 1650) or with DEP sampled from a non-catalyst car, although this finding was only after 48 h and also not 24 h after exposure. However they used a dose of 10 μg/cm^2 ^which is about 6 times less than the high concentration of 125 μg/ml used in the current study [8,9]. Furthermore no cell death was found by Baulig et al., which used 16BE14o- cells exposed to DEP (10 to 30 μg/cm^2^) for 24 h [47]. Bayram and co-workers also examined human bronchial epithelial cell cultures after DEP treatment with concentrations between 10 and 100 μg/ml and did not find any cellular damage or detachment of the cells [5].

Moreover a reduced sensitivity of the TJ in the triple cell co-cultures can be excluded. The TEER of mono- and the triple cell co-cultures showed a very similar behavior after a EDTA treatment which is known to open the TJ [48]. The TEER value was reduced time-dependently in both culture types. As already shown, the TEER values of the EC mono-cultures are generally higher compared to those of the triple cell co-cultures.

In this study the effect of DEP exposure on the TJ protein, occludin, was assessed for the first time in different mono-cultures of lung cells, including MDM, EC and MDDC. Additionally, the effects of DEP were studied using the combination of the three cell types, in the so-called triple cell co-culture system. It was not possible to assess the effects in the individual cells of the triple cell co-culture system, because there is no useful method known to separate the three cell types from the co-cultures system. However, isolating single cell types from the triple cell co-culture system might have a direct influence on the cell-cell junctions which might lead to altered results. Due to this separation problem, DEP exposure and their influence on the three cell types was assessed using single cell cultures. Using immunofluorescence methods, the characteristic belt-like structure of occludin, which was present in control EC cultures, was found to be reduced in the DEP treated EC cultures, supporting the data obtained with the TEER measurements. Additionally DEP treated MDM and MDDC mono-cultures exhibited a loss of occludin signals compared with the untreated control samples.

No dose-dependent reduction of occludin mRNA expression was found in MDM and MDDC. Lower doses of DEP (0.5-50 μg/ml) did not affect the expression whereas the highest DEP concentration (125 μg/ml) reduced the mRNA levels, although this difference was not significant. Interestingly, in untreated as well as in DEP treated mono-cultures of MDM, occludin was not only located at the cell border but also in the cytoplasm of the cells. In the *in vitro *triple cell co-culture model, the occludin protein is mainly present at the cell surface of MDM [28], suggesting that the localization of occludin is dependent on the presence of other cell types such as EC. It has also been shown in a kidney epithelial cell line that after the opening of TJ with the Ca^2+^-chelation method the occludin protein moved from the cell border towards the cytoplasm [48], indicating that cell-cell contacts are needed for the correct arrangement of occludin. Again, this is a reason for the examination of the expression level of occludin mRNA only in mono-cultures.

Interestingly, the expression levels of occludin mRNA and protein in EC did not change, although the TEER values decreased after DEP exposure. In fact, due to the expression level of the mRNA it is not possible to make a statement about the expression level of the respective protein because it is unclear if the mRNA is degraded or translated. The immunostaining in EC mono-cultures showed no change in the amount of protein, however the physiological arrangement of occludin was reduced after DEP treatment which could lead to an enhanced reduction of TEER. TEER is a commonly used marker for the epithelial integrity; the higher the TEER value is, the higher the epithelial integrity. Every epithelial cell (line) exhibits its own characteristic TEER values in untreated and standardized condition. It is proposed that the reduced integrity is due to the alteration in TJ disassembly rather than a reduction of the TJ protein occludin. When the TJ proteins are not placed in their physiological arrangement, the cell integrity can not be maintained. TJ are associated with the F-actin cytoskeleton and it has been shown that a reorganization of the F-actin cytoskeleton influences the formation and maturation of cell-cell contacts [42]. After cigarette smoke exposure to Calu-3 cells, amongst others, a displacement of occludin and ZO-1 was found and thus increasing ionic and macromolecular permeability. It was hypothesized that the loss of TJ integrity and barrier function was due to a decreased ZO-1:occludin association ratio. [34]. Another study using the Ca^2+ ^chelation method to open the TJ in MDCK, a kidney epithelial cell line, [48] supports the hypothesis that the opening of the TJ is not due to a reduced protein level but to a rearrangement of the TJ proteins. In control cultures, occludin was mainly found at the cell-cell contacts whereas after EGTA treatment the staining disappeared from the cell borders and was diffused closer to the membrane. In these experiments the TEER values reduced significantly. Inhibition of protein synthesis by addition of cycloheximide to the cell cultures did not influence the outcome of the Ca^2+ ^chelation experiment.

In the present study, the qualitative images of EC, MDM and MDDC showed an unaffected arrangement of the F-actin cytoskeleton after DEP treatment. Moreover, for the qRT-PCR the housekeeping gene β-actin was used, which showed a constant expression level throughout all experiments.

In pulmonary infections, bronchiolar or alverolar macrophages are one of the first cells responsible for clearing inhaled and deposited particles [46]. Furthermore macrophages are able to release pro-inflammatory cytokines such as TNFα and Interleukin 8. Mono-cultures of MDM and MDDC exposed to LPS showed a significantly higher TNFα response compared to the untreated control, whereas EC exposed to LPS showed no differences (data not shown). Focussing on TNFα, TNFα levels were determined from the supernatant in the *in vitro *triple cell co-culture model, which closely mimics the airway epithelium [27,37]. Due to a study in which it was shown that a TNFα induced drop in Caco-2 TEER was directly correlated with the increase in paracellular permeability, the TNFα levels were examined. This increase in Caco-2 TJ permeability was accompanied by the down-regulation of ZO-1 proteins and an alteration in the junction localization of those proteins [49]. No change in TNFα levels in the triple cell co-culture model was found, after exposure to the high dose of DEP (125 μg/ml) compared to the control. It is known that mono-cultures of macrophages show an enhanced pro-inflammatory reaction upon DEP exposure [50] therefore, the data from mono-cultures can not be directly compared to the triple co-culture model [36] The amount of macrophages in the triple co-culture model, which is similar to the amount in the human lung [30], is reduced compared to the mono-cultures and also there is the additional influence of the other two cell types. Additionally the mono-cultures of EC showed no pro-inflammatory reaction. This finding is in agreement with other publications obtained from animal studies: An *in vivo *study showed that long-term inhalation of a low-dose of DEP did not change TNFα release in alveolar macrophages of mice, but the release significantly decreased when they were exposed to a high dose [51]. Furthermore, cells from bronchoalveolar lavage fluid of rats instilled with DEP showed no enhanced TNFα release and the authors concluded that TNFα does not seem to be involved in the pathological changes associated with DEP exposure, in the time period studied [52].

## Conclusion

In summary, it was demonstrated that even after exposure to a high dose of DEP (125 μg/ml), the expression of occludin mRNA in 16HBE140- mono-cultures remained unaffected, while TEER decreased significantly and the local distribution of the occludin protein changed to an irregular pattern. The protein levels of occludin in MDM and MDDC were affected upon exposure to high DEP doses. Although the results were not significant, the mRNA levels of those cell types showed a tendency to be decreased after exposure to DEP. In contrast, no change in TEER was observed in the triple cell co-culture model and no pro-inflammatory reaction could be found. Further studies are required to understand the alterations of mRNA expression of the three cell types after stimulation or exposure to particles in the triple cell co-culture model to elucidate the influence of the cell-cell interactions between the different cell types. The TJ like structure could be shown between EC and MDM for the first time with TEM. By comparing the effects of the mono-cultures with an advanced triple cell co-culture model of the epithelial airway wall, the findings showed that the hazards of DEP in the triple cell co-culture model are less pronounced than in the mono-cultures. We hypothesize that there is a synergistic effect due to the interaction of the three cell types (epithelial cells, alveolar macrophages and dendritic cells) that reduce the adverse effects of DEP. The results encourage further study regarding the influence of DEP on other TJ proteins. There is a need that different cell types are included in cell culture models as this study has shown that co-culture models are better at simulating the real situation in the lung than mono-cultures.

## Methods

### 16HBE14o- mono-cultures

The 16HBE14o- bronchial epithelial cell line was used as described in earlier studies [30]. Briefly, 16HBe14o- cells (Passages 2.57-2.86) were cultured in MEM 1× medium, containing Earle's Salts, 25 mM HEPES, without L-Glutamine (Gibco BRL Life Technologies Invitrogen AG, Basel, Switzerland) supplemented with 1% L-Glutamine (LabForce AG, Nunningen, Switzerland), 1% penicillin/streptomycin (Gibco BRL), and 10% foetal calf serum (PAA Laboratories, Lucerna-Chem AG, Lucerne, Switzerland). The cells were grown in 25 cm^2 ^cell culture flasks (TPP, Trasadingen, Switzerland) which were treated with fibronectin coating solution containing bovine serum albumin, 0.1 mg/ml (Sigma, Fluka Chemie GmbH, Buchs, Switzerland) and 1% bovine collagen, Type I (BD Biosciences, Basel, Switzerland) and 1% human fibronectin (BD Biosciences) in LHC Basal Medium (Lucerna Chemie AG) and passaged in turns of every 3 and 4 days, respectively. For experimental cultures the cells were seeded on transparent BD Falcon cell culture inserts (surface area of 4.2 cm^2^, pores with 3.0 μm diameter, PET membranes for 6-well plates; BD Biosciences) treated with fibronectin coating solution containing bovine serum albumin (details are described above) and maintained for 7 days. Cultures were kept at 37°C in 5% CO_2 _humidified atmosphere.

### MDM and MDDC mono-cultures

MDM and MDDC were obtained from human peripheral blood monocytes as described by [53]. Briefly, peripheral blood monocytes were isolated from Buffycoat (Blood Donation Service, Bern, Switzerland) by density gradient centrifugation on Ficoll-Paque (Amersham Biosciences Europe GmbH, Otelfingen, Switzerland). Peripheral blood monocytes were re-suspended in RPMI 1640 supplemented with 1% L-Glutamine, 1% penicillin/streptomycin, and 10% heat-inactivated (pooled) human serum (Blood Donation Service, Bern, Switzerland), and then allowed to adhere for 90 min in 6-well plates (BD Biosciences,Basel, Switzerland). Non-adherent cells were washed away and adherent cells were cultured in RPMI 1640 medium, supplemented with 1% L-Glutamine, 1% penicillin/streptomycin, and 5% heat-inactivated (pooled) human serum. For the generation of MDDC, 34 ng/ml IL-4 (Sigma, Fluka Chemie GmbH, Buchs, Switzerland) and 50 ng/ml GM-CSF (R&D Systems, Oxon, UK) were added to the supplemented medium for 7-8 days. The MDM were only obtained in the supplemented medium for 7-8 days. The cell cultures were kept at 37°C in 5% CO_2 _humidified atmosphere in an incubator.

### Triple cell co-cultures

The co-cultures were prepared as previously described [30]. Briefly, 16HBE14o- cells were maintained on transparent BD Falcon cell culture inserts (surface area of 4.2 cm^2^, pores of 3.0 μm diameter, PET membranes for 6-well plates; BD Biosciences) treated with fibronectin coating solution containing bovine serum albumin (for details see above). Epithelial cells were cultured for 7-8 days before MDM were added on top of the epithelial monolayer and MDDC underneath the insert membrane. Before using the triple cell co-cultures, they were kept overnight in medium supplemented with 1% L-Glutamine, 1% penicillin/streptomycin, and 5% heat-inactivated (pooled) human serum at 37°C in 5% CO_2 _humidified atmosphere. The 16HBE14o- cells were kindly provided by Dr. Gruenert (University California, San Fransisco).

### Measurements of the transepithelial electrical resistance (TEER)

The TEER was measured with the Millicell-ERS system (MERS 000 01; Millipore AG, Volketswil, Switzerland) as described earlier [27,30,37]. Briefly, TEER was measured in the EC mono-cultures and in the triple cell co-cultures before and 24 h after DEP exposure. The mean of four measurements per insert was determined. The electrical resistance of insert membranes without cells was subtracted from all samples, and the resistance values were multiplied with the surface area of the inserts (4.2 cm^2^).

### DEP exposure of cell cultures and harvesting of cells

To determine the effects of different concentrations of DEP on the expression of TJ protein mRNA mono-cultures of MDM, MDDC and EC were exposed in suspension to standardised and characterised DEP (SRM Nr. 2975; National Department of Standards and Technology, Gaithersburg, USA). Prior to particle exposure the culture medium was removed and 2 ml of fresh, pre-warmed culture medium without serum, in which the DEP were diluted to the final concentrations of 0.5, 5, 50, and 125 μg/ml, respectively, was added to the cells and left for 24 h. Prior to incubation with cells, all DEP dilutions were sonicated for 2 min in order to minimize aggregation. Each experiment was repeated between 4 to 7 times.

### RNA isolation and RT-PCR

After exposure, the cells were removed from their substrate with a 25 cm cell scraper (Sarstedt, Sevelen, Switzerland), washed twice in phosphate buffered saline (PBS, 10 mM, pH 7.4: 130 mM NaCl, Na_2_HPO_4_, KH_2_PO_4_) and then stored in RNAlater (Qiagen, Basel, Switzerland) for the RNA isolation.

Total mRNA was extracted from the mono-cultures of MDDC, MDM and 16HBE14o- EC using the RNeasy Mini kit (Qiagen, Hombrechtikon, Switzerland) according to the manufacturer's protocol. Briefly, after centrifugation (30 s at 13000 × g) of the harvested cells, the supernatant was discharged and 600 μl buffer RLT (Qiagen, Hombrechtikon, Switzerland) with 6 μl beta-mercaptoethanol (Sigma-Aldrich Chemie GmbH, Buchs, Switzerland) was added to each sample for lysis, followed by homogenization using a glass douncer. Debris was pelleted by centrifugation (30 s at 13000 × g). A volume of 600 μl Ethanol (70%) was added to the cleared lysate before RNA was bound to the RNeasy membranes. 10 μl DNase I in 70 μl RDD buffer (Qiagen, Hombrechtikon, Switzerland) was added for 30 min at room temperature to remove traces of DNA. DNase and any contaminants were washed away with buffer RW1, and total RNA was eluted in RNAse-free water. Finally, obtained mRNA concentrations were spectrophotometrically determined using Nanodrop ND-1000 (NanoDrop Technologies). (NanoDrop, Witec AG, Littau, Switzerland). cDNA was synthesized by reverse transcription (RT) with 4 μg of total RNA using 2 μg Oligo (dT)15-primers (Promega, Wallisellen, Switzerland) and 4 U of Omniscript reverse transcriptase (Qiagen, Hombrechtikon, Switzerland) at 37°C for 1 h according to the manufacturer's protocol.

### Quantitative real-time-PCR (qRT-PCR)

The cDNA samples were analyzed by qRT-PCR using the self-designed primer pairs, specific for occludin mRNA (ordered by Mircrosynth, Balgach, Switzerland) (Table 1). For each investigated sample, 200 ng of the cDNA template was added to wells containing 12.5 μl of Sybr Green Jump Start Taq Ready Mix (Sigma Aldrich, Buchs, Switzerland) and 0.2 μl of both sense and antisense primers (to yield final concentrations of 0.6 μmol/l). RNAse free water was added up to the final volume of 25 μl. According to the manufacturer's protocol, two stages for the qRT-PCR were run. An initial step at 95°C for 2 min was followed by 40-cycle sequence of denaturation (15 s at 94°C), annealing (30 s at 56°C) and elongation (60 s at 72°C). (GeneAmp^® ^5700 Sequence Dedector, AB Applied Biosystems, Rotkreuz, Switzerland). For mRNA/cDNA expression analysis, cycling reports and melting curves were evaluated and the baseline cycle threshold was added. The reference housekeeping gene β-actin (Table 1) was used for normalisation of qRT-PCR data. Data were evaluated by the relative quantification method (2^-ΔCT^) as described by Livak and Schmittgen [54]. The qRT-PCR fragments were visualized by electrophoresis on a 2% (w/v) agarose (Axon, Baden-Dättwil, Switzerland) gel supplemented with 0.5 μg/ml ethidium bromide and were sequenced for occludin in the EC.

**Table 1:** Primer sequences for quantitative RT-PCR

**Gene**	**Sense and antisence sequences**	**Product length (bp)**
occludin	Left: 5'-GAA GCC AAA CCT CTG TGA GC-3'	229
	Right: 5'-GAA GAC ATC GTC TGG GGT GT-3'	

β-actin	Left: 5'-GGA CTT CGA GCA AGA GAT GG-3'	234
	Right: 5'-AGC ACT GTG TTG GCG TAC AG-3'	

### Cell fixation and labelling for laser scanning microscopy

Cells were labelled and fixed as described in detail (Blank et al., 2007). Antibodies were diluted in PBS as follows: mouse anti-human CD14 1:20 (Clone UCHM-1, C 7673, Sigma), mouse anti-human CD86 1:20 (Clone HB15e, 36931A, PharMingen, BD Biosciences), rabbit anti-occludin 1:50 (Clone Z-T22; Invitrogen, Basel Switzerland), goat anti mouse cyanine 2 1:50 (Chemicon, VWR International AG, Life Sciences, Lucerne, Switzerland), goat anti-mouse cyanine 5 1:50 (AP124S, Chemicon, VWR International AG, Life Sciences, Lucerne, Switzerland), goat anti rabbit cyanine 5 (Chemicon, VWR International AG, Life Sciences, Lucerne, Switzerland), Alexa 488 and phalloidin rhodamine 1:100 (R-415, Molecular Probes, Invitrogen AG, Basel, Switzerland).

### Confocal Laser scanning microscopy and image restoration

A Zeiss LSM 510 Meta with an inverted Zeiss microscope (Axiovert 200 M, Lasers: HeNe 633 nm, HeNe 543 nm, and Ar 488 nm) with a 63× objective lens (oil immersion, NA = 1.3) was used. For the detection of the TJ signals a negative sample containing only the secondary antibodies was scanned first and the detector gain was adjusted so that no fluorescent signal of the specific antibody (such as background) could be detected. The scans of the labelled samples were then acquired using the same detector settings. Image processing and visualization was done using IMARIS, a 3D multi-channel image processing software for confocal microscopic images (Bitplane AG, Zurich, Switzerland).

### Transmission electron microscopy

For TEM analysis, cells were fixed with 2.5% glutaraldehyde in 0.03 M potassium phosphate buffer, pH 7.4. The cells were postfixed with 1% osmium tetroxide in 0.1 M sodium cacodylate buffer and with 0.5% uranyl acetate in 0.05 M maleate buffer. Then cells were dehydrated in a graded series of ethanol and embedded in Epon. Ultrathin sections were cut and transferred on single slot grids (2 × 1 mm; Plano GMBH, Wetzlar, Germany), stained with uranyl acetate, counter-stained with lead citrate, and observed with a Philips 400 TEM at 60 kV (FEI Company Philips Electron Optics, Zurich, Switzerland).

### Determination of MDM in the triple co-culture model using TEM technique

It is not possible to define the MDM in the triple cell co-culture model only based on morphology. Therefore the differentiated MDM mono-cultures were exposed to 4 μg/ml gold NP with a diameter of 15 nm (British Biocell international, Cardiff, United Kingdom) for 18 h. Before adding the MDM to the triple cell co-culture model, the MDM mono-cultures were washed three times with pre-warmed medium to get rid of gold NP that were not internalized. The handling of the triple cell co-cultures is described in detail above. After 12 h, the cells were fixed as described above.

### Cytotoxicity

LDH was measured as an indicator of cell death by using a cytotoxicity detection kit (Roche, Roche Diagnostics, Rotkreuz, Schweiz) according to the manufacturer's manual. Briefly, supernatants of the EC mono-cultures and the triple cell co-cultures were collected from control, 0.5 and 125 μg/ml DEP exposed cultures after 24 h. The untreated cells (control) served as the negative control, cells treated with Trition-X-100 in PBS 1% served as a positive control. LDH activity was measured in a 96-well plate with three replicates of each group at an absorbance of 490 nm and with the reference wavelength at 630 nm.

### ELISA for determination of TNFα

After particle incubation for 24 h, supernatants of the epithelial mono-culture as well as from the triple cell co-cultures were collected from the upper and lower chamber and stored at -70°C. After centrifugation, TNFα was quantified by a commercially available DuoSet ELISA Development kit (R&D Systems, Catalogue Number: DY 210, Oxon, UK) according to the manufacturer's recommendations. Each sample was analysed in duplicates. The experiment was done in triplicate. The absorbance was read at 450 nm using an ELISA reader (SpectraMax 340 PC or Benchmark Plus Microplate Spectrophotometer (BioRad, Hempel Hempstead, UK)). The concentration of TNFα was determined and calculated by comparing the absorbance of the samples with standard recombinant human TNFα. For the positive control, mono- and triple cell co-cultures were also exposed to LPS (10 μl/ml). All values were normalized to the untreated control.

### Calcium Chelation

For those experiments, the previously described EC mono-cultures and the triple cell co-cultures were treated with EDTA at 37°. Untreated control cultures were also integrated. For the experimental cell cultures, the normal medium of the upper chamber was removed and fresh pre-warmed RPMI only supplemented with 2.0 mM EDTA was added at time point zero. For one hour, the TEER of the cell cultures were recorded every 15 min.

### Statistics

The results of ELISA, LDH and TEER measurements as well as the 2^-ΔCT ^values of the qRT-PCR are expressed as mean values with the standard deviation of the mean (SD). The statistical analysis was performed using SigmaStat for Windows (Version 3.10, Systat Software, Inc., Richmond, California, USA) statistical software. To compare more than two groups an ANOVA on Ranks was performed. Two groups were compared using a Mann-Whitney rank sum test, for comparison of before and after DEP exposure. In all cases p < 0.05 was considered to be significant.

## List of abbreviations

DEP: Diesel exhausted particle(s); DC: Dendritic cells in general; EC: Epithelial cell; EDTA: Ethylenediaminetetraacetic acid; LDH: Lactate dehydrogenase; LPS: Lipopolysaccharid; LSM: Confocal laser scanning microscopy; MDM: Monocyte derived macrophages; MDDC: Monocyte derived dendritic cells; NP: Nanosized particles; PM: Particulate matter; qRT-PCR: Quantitative real-time polymerase chain reaction; TEM: Transmission electron microscopy; TEER: Transepithelial electrical resistance; TJ: Tight junctions; TNFα: Tumor necrosis factor alpha. ZO-1: Zonula occludens 1.

## Competing interests

The authors declare that they have no competing interests.

## Authors' contributions

ADL: has done the acquisition, the analysis and the interpretation of data, and made a first draft of the manuscript. FB: participated in the design of the study, established the molecular methods and helped to draft the manuscript. OB: helped to interpret and evaluate the results of the quantitative real-time PCR and helped to draft the manuscript. PG: critically revised the manuscript and interpreted the data. BR: was the project leader, interpreted the data and composed the final version of the manuscript. All authors read and approved the final manuscript.

## Acknowledgements

The author thanks A. Stokes and B. Tschirren and B. Krieger for their excellent technical support. They thank K. Dobson for proofreading the manuscript. They are indepted to Dr. Gruenert for providing the human bronchiolar cell line 16HBE14o-.

This work was supported by the Swiss National Science Foundation, the Johanna Dürmüller-Bol Foundation, the Swiss Agency for the Environment and the Deutsche Forschungsgemeinschaft.
